# Hypoxia Promotes Prostate Cancer Aggressiveness by Upregulating EMT-Activator Zeb1 and SK3 Channel Expression

**DOI:** 10.3390/ijms21134786

**Published:** 2020-07-06

**Authors:** Fanny Bery, Sandy Figiel, Sana Kouba, Delphine Fontaine, Maxime Guéguinou, Marie Potier-Cartereau, Christophe Vandier, Roseline Guibon, Franck Bruyère, Gaëlle Fromont, Karine Mahéo

**Affiliations:** 1 INSERM, N2C UMR 1069, University of Tours, F-37032 Tours, France; fanny.bery@etu.univ-tours.fr (F.B.); sandy.figiel@gmail.com (S.F.); sana.kouba@etu.univ-tours.fr (S.K.); delphine.fontaine@etu.univ-tours.fr (D.F.); maxime.gueguinou@univ-tours.fr (M.G.); marie.potier-cartereau@univ-tours.fr (M.P.-C.); christophe.vandier@univ-tours.fr (C.V.); roseline.guibon@univ-tours.fr (R.G.); gaelle.fromont-hankard@univ-tours.fr (G.F.); 2 Department of Pathology, CHRU of Tours, CEDEX 9, F-37044 Tours, France; 3 Department of Urology, CHRU of Tours, CEDEX 9, F-37044 Tours, France; f.bruyere@chu-tours.fr

**Keywords:** prostate cancer, hypoxia, Zeb1, calcium, lipids

## Abstract

Hypoxia is a well-established feature of prostate cancer (PCa) and is associated with disease aggressiveness. The hypoxic microenvironment initiates multiple adaptive responses including epithelial-to-mesenchymal transition (EMT) and a remodeling of calcium homeostasis involved in cancer progression. In the present study, we identified a new hypoxia signaling pathway with a positive feedback loop between the EMT transcription factor Zeb1 and SK3, a Ca^2+^-activated K+ channel, which leads to amplifying store-operated Ca^2+^ entry. Zeb1 and SK3 channel were strongly upregulated by hypoxia both in vitro and ex vivo in organotypic cultures of human PCa. Taking into account the sensitivity of the SK3 channel to the membrane lipid composition, we identified lipids such as Ohmline (an alkyl ether lipid and SK3 inhibitor), linoleic acid (LA) and eicosapentaenoic acid (EPA) (fatty acids associated with indolent PCa), which were able to completely abrogate the hypoxia-induced changes in Zeb1 expression. Ultimately, better understanding of this new hypoxia-induced EMT pathway may allow to develop adjuvant therapeutic strategies, in order to control PCa aggressiveness and improve treatment outcomes.

## 1. Introduction

Hypoxia is known to contribute to cancer progression and is associated with resistance to treatment and metastatic dissemination [1]. In prostate cancer (PCa), the most common cancer type in men, hypoxia is a common feature that correlates with poor prognosis [2,3].

In most tumors, hypoxia leads to epithelial-to-mesenchymal transition (EMT), a process which allows cancer cells to acquire migratory and invasive characteristics, essentials for dissemination and distant metastases [4,5]. In fact, hypoxia has been shown to regulate the expression of EMT markers such as Snail or Zeb1 [6,7,8]. However, the intracellular signaling pathways that mediate hypoxia-induced EMT remain unclear. Among EMT markers, we previously demonstrated the clinical significance of Zeb1 in human PCa. Indeed, its expression is increased according to the different steps of PCa progression and is associated with decreased overall survival [9]. Zeb1 is a pivotal EMT transcription factor that promotes multidrug resistance, proliferation, and metastasis [4,10].

Hypoxia was found to initiate multiple adaptive responses, allowing cancer cells to survive in a hostile microenvironment [11]. Recent findings demonstrate the relationship between hypoxia and calcium (Ca^2+^) in different cancer types [12]. Ca^2+^ signaling is significantly remodeled in several cancer cell types, and gives them advantages for proliferation, migration, invasion, and resistance to apoptosis [13]. We previously demonstrated the pivotal role of the SK3 channel, a Ca^2+^-activated potassium channel, regulating Ca^2+^ entry in breast, colon, and prostate cancer aggressiveness [14,15,16]. SK3 has been shown to form complexes with Ca^2+^ channels, leading to an increase in cytosolic Ca^2+^ concentration in cancer cells and metastasis development. Interestingly, SK3 is located in cholesterol-enriched membrane nanodomains, also known as lipid rafts, and its function is dependent on the lipid environment [14]. We previously identified a fatty acid (FA) profile inversely associated with PCa aggressiveness and characterized by high levels of linoleic acid (LA) and eicosapentaenoic acid (EPA) [17]. Furthermore, we highlighted that these FAs exert their protective effects through a reduction of Ca^2+^ entry, involved in Zeb1 upregulation induced by TGFb, another EMT inducer [16]. In the present study, we report that hypoxia-induced Zeb1 is associated with activation of SK3 channel expression in PCa cells and subsequently affects their potential of aggressiveness. Moreover, and most importantly, the protective lipids identified in human PCa, namely LA and EPA, were found to totally reverse this process.

## 2. Results

### 2.1. Hypoxia Induces Zeb1 and SK3 Expression in Human Prostate Cancer Cells and Human Malignant Prostate Tissue

**Figure 1:**
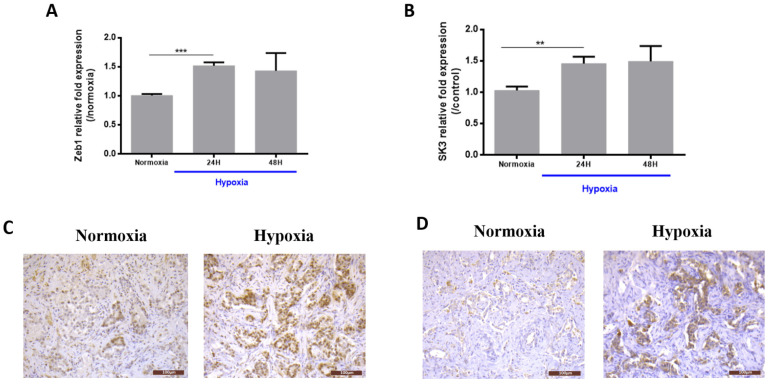
Expression of Zeb1 and SK3 channel under hypoxia conditions. (**A**) Zeb1 and (**B**) SK3 expression in PC3 cells. Cells were cultured under normoxic conditions for 24 h and then were cultured under hypoxia (1% O_2_) for 24 h or 48 h. qPCR results are normalized to normoxic conditions (*N* = 3; *n* = 3; ** *p* < 0.01; *** *p* < 0.001; Kruskal–Wallis, post-test: Dunn). (**C**) Zeb1 and (**D**) SK3 expression in human prostate cancer slices (Scale = 100 µm). Organotypic cultures were cultured under normoxia for 24 h and then were cultured under hypoxia (1% O_2_) for another 24 h. Five slices were obtained for each patient (*N* = 4 patients) to test the different conditions.

Human PCa cells PC3 were exposed to hypoxia for 24 h or 48 h. Zeb1 mRNA levels were increased by ~60% after hypoxia (24 h and 48 h) compared to normoxic conditions (Figure 1A). Since it was previously reported that *KCNN3* (the SK3 encoding gene) is a target gene of Zeb1 [16], we investigated whether the SK3 channel could also be regulated by hypoxia. Figure 1B shows that after 24 h and 48 h under hypoxia, SK3 mRNA is increased and interestingly at the same level as that of Zeb1 (by ~60%). 

We next investigated whether these findings could also be reproduced in clinical samples. Hypoxic conditions were tested in human malignant prostate tissue slices (*N* = 4 patients). Figure 1C,D shows representative immunohistochemistry pictures of Zeb1 and SK3 protein expression under normoxic and hypoxic conditions. Under normoxic conditions, cancer cells showed focal or absent Zeb1 expression, without significant SK3 staining. Hypoxia led to an increase in Zeb1 (4 cases out of 4) and SK3 (3 cases out of 4) expression, with a diffuse positive staining.

### 2.2. SK3 Channel Contributes to PCa Aggressiveness by Increasing Store-Operated Ca^2+^ Entry, Cell Migration, and Zeb1 Expression

**Figure 2:**
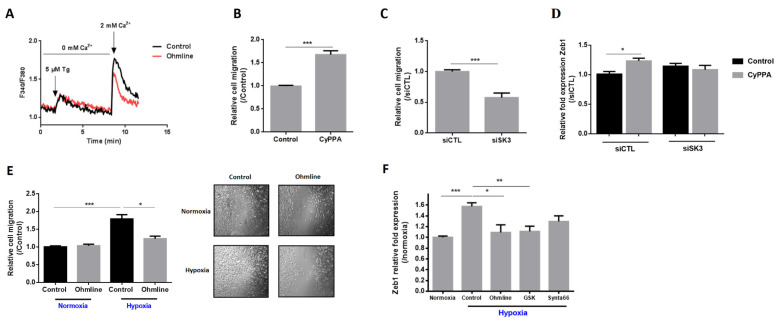
SK3 channel induces Zeb1 expression and cell migration under hypoxia conditions. (**A**) SOCE is inhibited by Ohmline (an SK3 inhibitor). Fluorescence measurements and relative fluorescence of Ca^2+^ entry after endoplasmic reticulum Ca^2+^ store depletion by thapsigargin (Tg) in PC3 cells pretreated for 48 h with Ohmline (1 µM). (**B**,**C**) SK3 channel promotes PC3 cell migration. (**B**) PC3 cells were treated with an SK3 activator, CyPPA (10 µM), and used for transwell migration assay performed for 24 h (*N* = 5; *n* = 2; *** *p* < 0.001; Mann–Whitney test). (**C**) PC3 cells were transfected with a siRNA directed against SK3 for 24 h and then used for transwell migration assay performed for 24 h (*N* = 3; *n* = 3; *** *p* < 0.001; Mann–Whitney test). (**D**) SK3 activator regulates Zeb1 expression. PC3 cells were transfected with a siRNA directed against SK3 for 24 h and then treated with CyPPA (10 µM) for another 24 h (**C**) (N = 4; *n* = 3; * *p* < 0.05; Kruskal–Wallis, post-test: Dunn). (**E**) SK3 regulates hypoxia-induced migration. PC3 cells were treated with an SK3 inhibitor, Ohmline (1 µM), and used for transwell (left) and wound-healing (right) migration assays performed for 24 h under normoxic or hypoxic conditions (*N* = 5; *n* = 2; * *p* < 0.05; *** *p* < 0.001; Kruskal–Wallis, post-test: Dunn). (**F**) Hypoxia-induced Zeb1 expression is abolished by channel inhibitors. PC3 cells were treated with Ohmline, GSK7975A, and Synta66 (inhibitors of TRP and Orai channels) for 48 h and cultured under hypoxia (1% O_2_) for the last 24 h (N = 3; *n* = 3; * *p* < 0.05; ** *p* < 0.01; *** *p* < 0.001; Kruskal–Wallis, post-test: Dunn).

In non-excitable cells, Ca^2+^ entry from extracellular medium is mainly supported by a capacitive mechanism, also known as store-operated Ca^2+^ entry (SOCE), which is mediated by store-operated Ca^2+^ channels [18]. Treatment of PC3 cells with Ohmline (1-O-hexadecyl-2-O-methyl-sn-glycero-lactose), an alkyl ether lipid inhibitor of the SK3 channel, significantly decreased the amplitude of SOCE (by ~20%) (Figure 2A), demonstrating that this Ca^2+^ entry is regulated by the SK3 channel in PC3 cells. 

To determine whether the SK3 channel has a pivotal role in cell migration as already reported in PCa cells and in other cell types [14,16], we performed transwell migration assays with cells treated by a pharmacological SK3 activator (CYPPA) or with siRNA-SK3-transfected cells. As expected, cell migration was strongly increased by CYPPA (+69%) (Figure 2B). In contrast, the suppression of SK3 showed significantly reduced migratory ability of cancer cells (−43%) (Figure 2C). These findings demonstrate that SK3 contributes to PCa aggressiveness by increasing SOCE and PCa cancer cell migration. We then analyzed the effect of CyPPA on Zeb1 expression in PC3 cells transfected or not with siSK3. CyPPA treatment led to an increase in the Zeb1 mRNA level by 23% under si-control conditions, and had no effect on Zeb1 expression in siSK3-transfected cells. This finding demonstrates a direct effect of SK3 on Zeb1 expression.

As shown above, the SK3 channel was induced by hypoxia and was able to increase cell migration in normoxia. Therefore, we studied whether SK3 plays a role in hypoxia-induced migration. Cells were treated with Ohmline and used for transwell and wound-healing migration assays performed for 24 h (+/− Ohmline). Hypoxia strongly induced cell migration (+78%) (Figure 2E). The SK3 inhibitor Ohmline was able to completely abrogate the hypoxia-induced migration.

As SK3 has been shown to form complexes with Ca^2+^ channels, leading to an increase in cytosolic Ca^2+^ concentrations, we wondered whether the mechanism involved in hypoxia-induced Zeb1 expression depends on Ca^2+^. Figure 2F shows that the upregulation of Zeb1 by hypoxia was inhibited by Ca^2+^ channel inhibitors GSK7975A and Synta66 (inhibitors of TRP and Orai channels, respectively). Interestingly, the SK3 inhibitor Ohmline was also able to completely abrogate the effect of hypoxia on Zeb1 expression. All together, these results suggest that hypoxia induces a positive feedback loop between Zeb1 and the SK3 channel in a pathway that leads to an increase of Ca^2+^ entry. 

### 2.3. LA and EPA Lipids Totally Block the Expression of Zeb1 and SK3 Induced by Hypoxia 

We recently reported that LA and EPA, FAs associated with indolent PCa, reduced SOCE in PCa cells [16]. Here, we hypothesized that these lipids may modulate Zeb1 and SK3 gene expression induced by hypoxia. 

**Figure 3:**
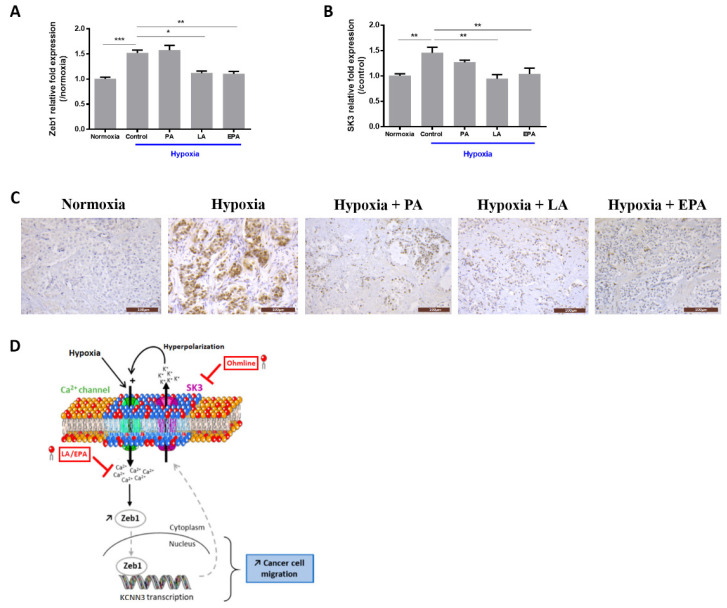
Linoleic acid (LA) and eicosapentaenoic acid (EPA) inhibit hypoxia-induced Zeb1 and SK3 expression. (**A**,**B**) FA effects on hypoxia-induced Zeb1 and SK3 expression. PC3 cells were treated with FAs for 48 h and the last 24 h cultured under hypoxia (1% O_2_). qPCR results are expressed in 2^−ΔΔCt^, (*N* = 3; *n* = 3; * *p* < 0.05; ** *p* < 0.01; *** *p* < 0.001; Kruskal–Wallis, post-test: Dunn’s test). (**C**) Zeb1 expression in human prostate cancer slices (Scale = 100 µm). Organotypic cultures were treated with FAs (60 µM) and cultured under normoxia for 24 h and then were cultured (with FAs) under hypoxia (1% O_2_) for another 24 h; Zeb1 nuclear expression increased under hypoxia conditions. After palmitic acid (PA) treatment, most of the cancer cells (present in the upper right area) were still positive for Zeb1, whereas after treatment with either LA or EPA, only rare Zeb1 positive cancer cells were observed. Five slices were obtained for each patient (*N* = 4 patients) to test the different conditions. (**D**) Proposed schematic model for a positive feedback loop leading to prostate cancer (PCa) aggressiveness and inhibited by LA and EPA based on our results. In fact, hypoxia induces the transcription factor Zeb1 known to be regulated by calcium. Zeb1 targets the *KCNN3* gene encoding for SK3. At the plasma membrane, the SK3 channel allows an increase in calcium entry by hyperpolarization of the plasma membrane. By incorporating Ohmline, LA, and EPA into the membrane, we observed that they inhibit this signaling pathway induced by hypoxia.

As observed in Figure 3, hypoxia treatment failed to induce Zeb1 and SK3 expression in LA- and EPA-supplemented cells (Figure 3A,B). To assess the clinical relevance of these results, human PCa slices were treated with these FAs for 48 h and for the last 24 h they were cultured under hypoxia (N = 4 patients). Treatment by LA and EPA prevented either totally (LA) or partially (EPA) Zeb1 expression induced by hypoxia (Figure 3C), whereas palmitic acid (PA) had no effect. These data suggest a pivotal role for EPA and LA to prevent hypoxia-induced EMT.

## 3. Discussion

Hypoxia is a common feature in the microenvironment of solid tumors, and it promotes cancer cell survival and aggressiveness [1,19]. Here, we report that hypoxia induces the EMT transcription factor Zeb1 through a Ca^2+^ -dependent mechanism involving SK3, a Ca^2+^-activated K^+^ channel sensitive to lipids. The data in human PCa samples strongly emphasized the clinical consistency of our observations. The lipids tested in this study were selected because of their potential clinical interest, and include Ohmline, a synthetic alkyl ether lipid with antimetastatic effects [14,15], as well as LA and EPA, FAs inversely associated with PCa aggressiveness [17]. 

Although EMT and hypoxia were initially considered as separate events promoting invasion and metastasis, several recent studies have shown that the signaling pathways are interrelated [20]. Among the potential mediators of hypoxia-induced EMT, a role of hypoxia-inducible factor 1α (HIF-1α) has been reported. HIF-1α was found to directly regulate Zeb1, which has four hypoxia-response element (HRE) sites on its proximal promoter [7]. An HIF-1α/Zeb1 signaling axis initiated by hypoxia has been described in several types of cancer cells [21,22].

Interactions between hypoxia and Ca^2+^ modulators, including channels, pumps, and sensors have been recently highlighted [12]. On the one hand, the expression of Ca^2+^ regulating proteins is controlled by HIF-1 and, on the other hand, these proteins are involved in the regulation of HIF-1 activity [12]. In PCa, TRPM8 promotes HIF-1α protein levels by suppression of RAK1-mediated HIF-1α ubiquitination [23]. Here, we report for the first time that the expression of SK3 is increased by hypoxia. Recently, we demonstrated that SK3 expression was upregulated by TGFβ, a potent inducer of EMT, and that *KCNN3* (the SK3 encoding gene) is a target gene of Zeb1 [16]. These data suggest that the SK3 channel is upregulated by factors of the tumor microenvironment, including TGFb and hypoxia, which may explain its differential expression in tumor and non-tumor tissues [14].

In the present study, we provide evidence that hypoxia-induced Zeb1 expression was inhibited by either Ohmline (SK3 inhibitor) or Ca^2+^ channel inhibitors. SK3 has been shown to form complexes with Ca^2+^ channels, leading to an increase in cytosolic Ca^2+^ concentration promoting breast and colon cancer cell migration [14,15]. Here, we showed that the SK3 channel regulated SOCE and migration induced by hypoxia in PCa cells. Taken together and as summarized in Figure 3D, our results suggest a positive feedback loop between Zeb1 and SK3 expression under hypoxic conditions, leading to Ca^2+^ entry amplification (by hyperpolarizing the membrane). This model requires further experiments in a future study to specify the sequence of events and actors involved in the Ca^2+^ regulation.

Calcium signaling plays a major role in several events driving cancer progression, including proliferation, cell migration, angiogenesis, and apoptosis [24]. An increase of Ca^2+^ entry in malignant tissues compared with benign tissues was also demonstrated [25]. Recently, we reported higher intracellular Ca^2+^ entries in human tissue samples from high-risk PCa compared with indolent tumors [26]. Our findings raise the hypothesis that Ca^2+^ homeostasis could play a pivotal role in PCa progression. 

The location of SK3 in cholesterol-enriched membrane nanodomains, also known as lipid rafts, with a function dependent on the lipid environment, provides an opportunity for the control of prostate cancer aggressiveness. Indeed, by incorporating a lipid such as Ohmline into nanodomains, we observed that it delocalizes the SK3 channel outside lipid nanodomains and reduces SOCE-dependent cell migration [14]. We recently reported that LA and EPA reduced Ca^2+^ entry involved in Zeb1 regulation by TGFb, leading to cancer cell migration [16]. Here, we report that the expression of both Zeb1 and SK3 are strongly inhibited by LA and EPA supplementation. Since LA and EPA administration in humans has been shown to be well tolerated, with any toxic effect [27], the present study reinforces their potential applications as an adjuvant treatment to control PCa aggressiveness. 

## 4. Materials and Methods

### 4.1. Human Tissue Slice Cultures 

Prostatic tissue samples, including malignant and normal tissues, were obtained from 4 patients undergoing radical prostatectomy for PCa. Patients were 66 to 75 years old, with PCa grade ISUP1 (*n* = 1), ISUP2 (*n* = 2), or ISUP 5 (*n* = 1). All tumors were staged pT2. Written informed consents were obtained from patients following the requirements of the medical ethics committee of our institution (Comité de Protection des Personnes (CPP) de Tours—Région Centre Ouest I) (ethics code DC-2014-2045) (years between 2003 and 2018). Immediately after surgery, 4 to 5 mm samples were dissected aseptically and cut with a vibratome into 6–10 slices per sample, as previously described [26]. Slices were incubated with DMEM medium supplemented with 10% fetal bovine serum (FBS), 1% penicillin–streptomycin, 1nM dihydrotestosterone, and placed in a 37 °C-humidified incubator with 5% CO_2_ in normoxia or in a hypoxia chamber at 1% O_2_.

### 4.2. Immunohistochemistry 

Tissue slices or cells were fixed in 10% formalin, embedded in paraffin, and cut in serial 3 µm sections. One section was stained with hematoxylin-eosin-saffron (HES), and the other sections were deparaffinized, rehydrated, and heated in citrate buffer pH 6 for antigenic retrieval. After blocking for endogenous peroxidase with 3% hydrogen peroxide, primary antibodies were incubated for 1 h, including Zeb1 (Abnova, Taoyuan, Taiwan, dilution 1/500) and SK3 (LSBio, Seattle, WA, USA, dilution 1/200). Immunohistochemistry was then performed using the streptavidin–biotin–peroxydase method with diaminobenzidine (DAB) as the chromogen (Kit LSAB, Dakocytomation, Glostrup, Denmark). Slides were finally counterstained with hematoxylin. Positive staining was expressed as a percentage of total cancer cells. 

### 4.3. Cell Lines and Products

Human PCa PC3 (CRL-1435) cell line (American Type Culture Collection (ATCC)) was received in 2016–2018. This cell line was tested and authenticated by DNA fingerprinting by the ATCC. After reception, cells were amplified in order to make a large reserve of cryopreserved cells. Every 3 months, a new cryopreserved bulb was thawed and used for this study. PC3 cells were cultured in RPMI medium (BE12-702F, Lonza, Levallois-Perret, France), supplemented with 5% FBS (CVFSVF0001, Eurobio, Les Ulis, France) and 1% (*v/v*) penicillin–streptomycin in a 37 °C-humidified incubator, 5% CO_2_.

LA (L1876), EPA (17266), and PA (P5177) were from Sigma-Aldrich (Saint-Quentin-Fallavier, France). Calcium channel inhibitors GSK7975A (GLXC03243) and Synta66 (GLXC03244) were from GLIXX Laboratories INC (Hopkinton, MA, USA). The SK3 activator, CyPPA, was purchased from Sigma-Aldrich (Saint-Quentin-Fallavier, France). 1-Ohexadecyl-2-O-methyl-sn-glycero-3-lactose (Ohmline) was synthetized as previously described [28]. 

### 4.4. RNA Extraction and Quantitative Real-Time PCR

Total RNAs from cultured cells were extracted using the Nucleopsin RNA kit (Macherey-Nagel, Hoerdt, France). To obtain cDNA at 50 ng/µL, RNAs were reverse transcribed with an RT kit (PrimeScriptTM RT Reagent, Perfect Real Time, Takara, Saint-Quentin-en-Yvelines, France). The reaction progressed in a LightCycler 480 (Roche Applied Science, Meylan, France) at 37 °C for 17 min followed by 85 °C for 5 min. For each reaction, SYBR Green mix (RR420L, Takara) was mixed with specific primers (0.5 µM, see Supplementary Materials) and cDNA at 50 ng/µL. Relative levels of mRNA were calculated according to the DDCT method relative to the housekeeping gene HPRT and cyclophilin A. Primers used for quantitative real-time PCR are available in Table S1, Supplementary Materials.

### 4.5. Cytosolic Calcium Measurements 

Cytosolic Ca^2+^ concentrations were studied using the ratiometric fluorescent dye Fura-2-AM from Thermo Fischer Scientific (Illkirch, France) (1H, 5 µM). A SOCE protocol was followed: cells were incubated in a physiological saline solution (PSS) without Ca^2+^, whose composition is detailed in the Supplementary Materials (Table S2). After a short stabilizing time, PC3 cells were treated by a SERCA pump inhibitor, thapsigargin (Tg 5 µM). After total endoplasmic reticulum-Ca^2+^ depletion, PSS with 2 mM CaCl_2_ (2Ca) was added. Fluorescence emission was measured at 510 nm with an excitation light at 340 and 380 nm. Analyses were performed using SoftMax Pro Software (5.4.6 version, Molecular Devices, San Jose, CA, USA).

### 4.6. Migration Assays 

Transwell assays were performed in 24-well plates receiving 8 µm pore size cell culture inserts (353097, Falcon, Boulogne-Billancourt, France). 

For treated cells with CyPPA, 30,000 cells were seeded in the upper compartment containing growth medium (5% FBS +/− CyPPA 10 µM) and allowed to migrate through the pores to the other side of the membrane for 24 h. The lower compartment was also filled with growth medium (10% FBS +/− CyPPA 10 µM). 

For siRNA-SK3-transfected cell migration, 24 h after transfection, 60,000 cells were seeded with a 5% to 10% FBS gradient in upper and lower compartments, respectively. After 24 h, migrated cells to the bottom side were fixed, stained, and automatically counted. 

For Ohmline treatment, 60,000 cells were seeded with a 5% to 10% FBS gradient (+/− Ohmline 1 µM) in upper and lower compartments. Migration assay was performed for 24 h under normoxic and hypoxic conditions.

Wound-healing assays were performed in 24-well plates (2515083, Falcon, Boulogne-Billancourt, France). For these assays, 80,000 cells were seeded and incubated at 37 °C for 24 h under normoxic conditions. Once the scar was made, cells were placed under normoxia or hypoxia for another 24 h. 

### 4.7. Transfection Assays 

In all, 200,000 cells/well were seeded in growth medium in a 6-well plate and incubated at 37 °C overnight. After 24 h, cells were transfected with a free-serum opti-MEM medium containing a mixture of Lipofectamine RNAimax (10514953, Fisher Scientific TM, Illkirch, France) and 20 nM of siRNA. Transfected cells were incubated at 37 °C for 6 h. The medium was then removed and replaced with a fresh growth medium. siRNA used in this study are available in Table S3 (Supplementary Materials). For CyPPA experiments, cells were treated after 24 h of transfection with CyPPA (10 µM) for another 24 h. 

### 4.8. Statistical Analysis

Statistical analysis was carried out using the Mann–Whitney test and Kruskal–Wallis one-way ANOVA followed by Dunn’s test (as indicated in figure legends). All results are expressed as mean +/− SEM.

## 5. Conclusions

The present study identified an original signaling pathway associated with increased PCa aggressiveness, promoted by hypoxia and leading to Zeb1 and SK3 induction through an increase of Ca^2+^ entry. LA and EPA supplementation strongly inhibited several steps of this process, which underlines their therapeutic potential as adjuvant for cancer treatments.

## Supplementary Materials

Supplementary materials can be found at https://www.mdpi.com/1422-0067/21/13/4786/s1.

## Abbreviations

EMT	Epithelial-to-mesenchymal transition
EPA	Eicosapentaenoic acid
HIF-1α	Hypoxia-inducible factor 1α
HRE	Hypoxia-response element
FA	Fatty acid
LA	Linoleic acid
PA	Palmitic acid
PCa	Prostate cancer
SOCE	Store-operated Ca2+ entry

## References

[1] Harris A.L. (2002). Hypoxia—A key regulatory factor in tumour growth. Nat. Rev. Cancer.

[2] Ragnum H.B., Vlatkovic L., Lie A.K., Axcrona K., Julin C.H., Frikstad K.M., Hole K.H., Seierstad T., Lyng H. (2015). The tumour hypoxia marker pimonidazole reflects a transcriptional programme associated with aggressive prostate cancer. Br. J. Cancer.

[3] McKeown S.R. (2014). Defining normoxia, physoxia and hypoxia in tumours-implications for treatment response. Br. J. Radiol..

[4] Lamouille S., Xu J., Derynck R. (2014). Molecular mechanisms of epithelial-mesenchymal transition. Nat. Rev. Mol. Cell Biol..

[5] Zhang P., Wei Y., Wang L., Debeb B.G., Yuan Y., Zhang J., Yuan J., Wang M., Chen D., Sun Y. (2014). ATM-mediated stabilization of ZEB1 promotes DNA damage response and radioresistance through CHK1. Nat. Cell Biol..

[6] Azimi I., Milevskiy M.J.G., Kaemmerer E., Turner D., Yapa K.T.D.S., Brown M.A., Thompson E.W., Roberts-Thomson S.J., Monteith G.R. (2017). TRPC1 is a differential regulator of hypoxia-mediated events and Akt signalling in PTEN-deficient breast cancer cells. J. Cell Sci..

[7] Zhang W., Shi X., Peng Y., Wu M., Zhang P., Xie R., Wu Y., Yan Q., Liu S., Wang J. (2015). HIF-1α Promotes Epithelial-Mesenchymal Transition and Metastasis through Direct Regulation of ZEB1 in Colorectal Cancer. PLoS ONE.

[8] Choi B.-J., Park S.-A., Lee S.-Y., Cha Y.N., Surh Y.-J. (2017). Hypoxia induces epithelial-mesenchymal transition in colorectal cancer cells through ubiquitin-specific protease 47-mediated stabilization of Snail: A potential role of Sox9. Sci. Rep..

[9] Figiel S., Vasseur C., Bruyere F., Rozet F., Maheo K., Fromont G. (2017). Clinical significance of epithelial-mesenchymal transition markers in prostate cancer. Hum. Pathol..

[10] Sánchez-Tilló E., Siles L., de Barrios O., Cuatrecasas M., Vaquero E.C., Castells A., Postigo A. (2011). Expanding roles of ZEB factors in tumorigenesis and tumor progression. Am. J. Cancer Res..

[11] Semenza G.L. (2013). HIF-1 mediates metabolic responses to intratumoral hypoxia and oncogenic mutations. J. Clin. Investig..

[12] Azimi I. (2018). The interplay between HIF-1 and calcium signalling in cancer. Int. J. Biochem. Cell Biol..

[13] Monteith G.R., Prevarskaya N., Roberts-Thomson S.J. (2017). The calcium–cancer signalling nexus. Nat. Rev. Cancer.

[14] Chantome A., Potier-Cartereau M., Clarysse L., Fromont G., Marionneau-Lambot S., Gueguinou M., Pages J.-C., Collin C., Oullier T., Girault A. (2013). Pivotal Role of the Lipid Raft SK3-Orai1 Complex in Human Cancer Cell Migration and Bone Metastases. Cancer Res..

[15] Guéguinou M., Harnois T., Crottes D., Uguen A., Deliot N., Gambade A., Chantôme A., Haelters J.P., Jaffrès P.A., Jourdan M.L. (2016). SK3/TRPC1/Orai1 complex regulates SOCE-dependent colon cancer cell migration: A novel opportunity to modulate anti-EGFR mAb action by the alkyl-lipid Ohmline. Oncotarget.

[16] Figiel S., Bery F., Chantôme A., Fontaine D., Pasqualin C., Maupoil V., Domingo I., Guibon R., Bruyère F., Potier-Cartereau M. (2019). A Novel Calcium-Mediated EMT Pathway Controlled by Lipids: An Opportunity for Prostate Cancer Adjuvant Therapy. Cancers (Basel).

[17] Figiel S., Pinault M., Domingo I., Guimaraes C., Guibon R., Besson P., Tavernier E., Blanchet P., Multigner L., Bruyère F. (2018). Fatty acid profile in peri-prostatic adipose tissue and prostate cancer aggressiveness in African–Caribbean and Caucasian patients. Eur. J. Cancer.

[18] Prevarskaya N., Skryma R., Shuba Y. (2011). Calcium in tumour metastasis: New roles for known actors. Nat. Rev. Cancer.

[19] Hill R.P., Bristow R.G., Fyles A., Koritzinsky M., Milosevic M., Wouters B.G. (2015). Hypoxia and Predicting Radiation Response. Semin. Radiat. Oncol..

[20] Tam S.Y., Wu V.W.C., Law H.K.W. (2020). Hypoxia-Induced Epithelial-Mesenchymal Transition in Cancers: HIF-1α and Beyond. Front. Oncol..

[21] Joseph J.V., Conroy S., Pavlov K., Sontakke P., Tomar T., Eggens-Meijer E., Balasubramaniyan V., Wagemakers M., den Dunnen W.F.A., Kruyt F.A.E. (2015). Hypoxia enhances migration and invasion in glioblastoma by promoting a mesenchymal shift mediated by the HIF1α–ZEB1 axis. Cancer Lett..

[22] Zhu J., Huang Z., Zhang M., Wang W., Liang H., Zeng J., Wu K., Wang X., Hsieh J.-T., Guo P. (2018). HIF-1α promotes ZEB1 expression and EMT in a human bladder cancer lung metastasis animal model. Oncol. Lett..

[23] Yu S., Xu Z., Zou C., Wu D., Wang Y., Yao X., Ng C.-F., Chan F.L. (2014). Ion channel TRPM8 promotes hypoxic growth of prostate cancer cells via an O2-independent and RACK1-mediated mechanism of HIF-1α stabilization. J. Pathol..

[24] Clapham D.E. (2007). Calcium signaling. Cell.

[25] Stewart T.A., Yapa K.T.D.S., Monteith G.R. (2015). Altered calcium signaling in cancer cells. Biochim. Biophys. Acta.

[26] Figiel S., Pasqualin C., Bery F., Maupoil V., Vandier C., Potier-Cartereau M., Domingo I., Guibon R., Bruyere F., Maheo K. (2019). Functional Organotypic Cultures of Prostate Tissues. Am. J. Pathol..

[27] Saini R.K., Keum Y.-S. (2018). Omega-3 and omega-6 polyunsaturated fatty acids: Dietary sources, metabolism, and significance—A review. Life Sci..

[28] Girault A., Haelters J.-P., Potier-Cartereau M., Chantome A., Pinault M., Marionneau-Lambot S., Oullier T., Simon G., Couthon-Gourvès H., Jaffrès P.-A. (2011). New alkyl-lipid blockers of SK3 channels reduce cancer cell migration and occurrence of metastasis. Curr. Cancer Drug Targets.

