# Supplementary figures and images for: Hypoxia Promotes Prostate Cancer Aggressiveness by Upregulating EMT-Activator Zeb1 and SK3 Channel Expression
Source: Int J Mol Sci. 2020 Jul 6;21(13):4786. doi: 10.3390/ijms21134786 (PMC7369999; doi:10.3390/ijms21134786)

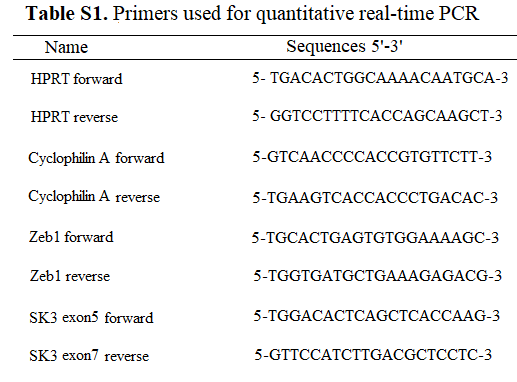

Supplement: Supplementary file 1 [file ijms-21-04786-s001.zip › Table S1.tif]

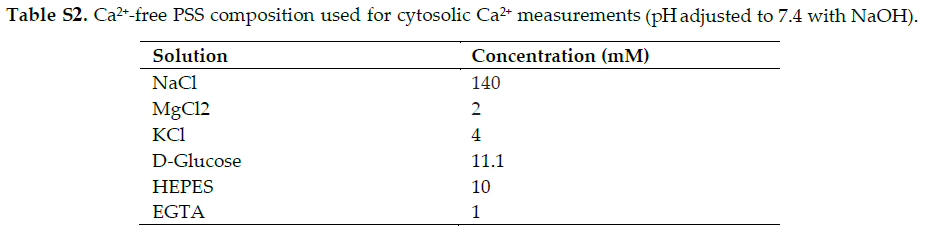

Supplement: Supplementary file 1 [file ijms-21-04786-s001.zip › Table S2.tif]

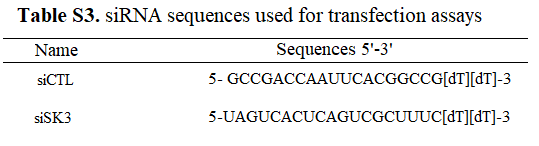

Supplement: Supplementary file 1 [file ijms-21-04786-s001.zip › Table S3.tif]
